# Patterns in Cardiovascular Risk Factors Stratified by Age and Sex in a Large German Population

**DOI:** 10.1016/j.jacadv.2026.103159

**Published:** 2026-08-19

**Authors:** Julia Christina Lueg, Daniel Schulze, Henrike Radefeld, Anne-Kathrin Collisi, Verena Stangl, Anna Brand, Veronika Zach

**Affiliations:** a Deutsches Herzzentrum der Charité, Department of Cardiology, Angiology and Intensive Care Medicine, Berlin, Germany; b Charité – Universitätsmedizin Berlin, Corporate Member of Freie Universität Berlin and Humboldt-Universität zu Berlin, Berlin, Germany; c DZHK (German Centre for Cardiovascular Research), Berlin, Germany; d Charité – Universitätsmedizin Berlin, Corporate Member of Freie Universität Berlin and Humboldt- Universität zu Berlin, Institute of Biometry and Clinical Epidemiology, Berlin, Germany; e ias PREVENT GmbH, Berlin, Germany; f Clinic of Internal Medicine - Cardiology and Conservative Intensive Care Unit, Vivantes Humboldt-Krankenhaus, Berlin, Germany; g Universitätsklinik für Innere Medizin, Medizinische Universität Graz, Graz, Österreich

**Keywords:** cardiovascular risk factors, menopause, prevention, sex-specific, working population

## Abstract

**Background:**

Despite growing evidence of sex-specific differences in cardiovascular (CV) disease, sex-specific prevention strategies are lacking.

**Objectives:**

This study aims to examine CV risk factors according to age and sex in a German working population.

**Methods:**

In collaboration with ias PREVENT, a provider of medical checkups for employees of German companies, a data set of 40,704 checkups was evaluated. The cohort was stratified by sex and age into 6 groups: women and men <45 years, between 45 and 55 years, and >55 years, respectively. The study analyzed the distribution of CV risk factors with a focus on sex-specific differences.

**Results:**

The average age of the cohort was 49 years (25% women). The cohort exhibited overall low burden of CV risk factors. The prevalence of hypertension, elevated low-density lipoprotein, and diabetes was 18.4%, 39.5%, and 5.3% in women compared to 30.7%, 58.7%, and 5.7% in men. In the group ages 45 to 55 years old, a higher proportion of women than men smoked. Obesity prevalence was higher in women than in men starting at the age of 50. Diabetes prevalence was higher in women than men under 45 but reversed in men above 55 years.

**Conclusions:**

The study underscores the need for sex- and age-specific CV prevention in the working environment. Participants demonstrated escalating CV risk, particularly with increasing age (45-55 years). This highlights the need for targeted measures for this age group, including the menopausal phase in women.

In recent years, growing evidence elucidated the existence of sex-specific differences in the prevalence, incidence, and outcomes of cardiovascular (CV) disease.^1^, ^2^, ^3^ In the context of individualized, patient-centered medicine, the stratification of sex-specific differences is crucial. The perimenopausal window, in particular, represents a critical transition associated with a significant change of CV risk in women compared to men.^3^ This highlights the need for sex-specific, individualized prevention. Due to a substantial lack of data largely driven by the underrepresentation of women in most of CV clinical trials, different prevention strategies for men and women are still sparse.^4^

In the light of the increasing prevalence of major CV risk factors such as obesity, diabetes, and arterial hypertension and the mounting burden on global health care systems,^5^, ^6^, ^7^ current guidelines advocate for a population-centered approach to preventive strategies, with particular emphasis on the working environment.^4^ Indeed, CV diseases, associated with high morbidity and mortality, can result in a considerable rise in direct and indirect costs for companies, leading to a loss of working power and efficiency.^8^ Therefore, prevention has become increasingly important to ensure sufficient capacity of the industry and global health systems.

Recent data from the Global Cardiovascular Risk Consortium has demonstrated the importance of optimizing CV risk factors already in middle age.^9^ In both women and men, control of arterial hypertension between the ages of 55 and 60 was associated with the greatest gain in life years free from CV disease compared to uncontrolled hypertension.^9^ Of note, when comparing patients with none of the 5 CV risk factors (arterial hypertension, hyperlipidemia, under- and overweight or obesity, diabetes, and smoking at age 50) to those presenting with all of them, the gain in life years was numerically greater in women than in men.^9^ Current data also show that, even though overall prevalence of ischemic heart disease is lower in women, affected women experience higher mortality rates than men.^10^

A recent German study has indicated a decline in the overall life expectancy of men and women.^11^ This trend is more pronounced among men. For men, the difference in life expectancy between Germany and Spain has more than doubled (from 1.0 to 2.1 years), whereas for women, it has increased from 2 years in 2006 to 3 years in 2019.^11^ This difference appears to be largely driven by an increasing incidence of CV mortality among the elderly population and underscores the necessity for enhanced primary health care and CV prevention measures.^11^

The present study was carried out in collaboration with ias PREVENT, a company dedicated to health promotion among working individuals in the last 40 years. Using a large data set, this study aims to illustrate the distribution and age differences of classical CV risk factors in the working population. The data shall serve as both an epidemiological population reference and a source for identifying relevant sex differences across age groups. Particular emphasis will thus be placed on sex-specific CV risk patterns and their potential burden. The data may help identify specifically vulnerable subgroups and contribute to the development of targeted preventive strategies within the daily working framework.

## Methods

The data presented in this study were derived from a data set provided by ias PREVENT and their preventive medical checkups using a standardized questionnaire. A total of 75,611 checkups were carried out in the period from 2011 to 2022. The number of examinations per subject varied greatly, ranging from one examination within 10 years to one subject undergoing 20 examinations. Ten duplicates and 30,917 follow-up examinations (46.17%) were excluded from the study. The final cohort, therefore, consisted of 40,704 individual participants.

## Outcome assessments

Given the wide variation in the number of visits per participant, only the first visits were included in this analysis. The use of data from the initial visits is intended to provide the most representative CV risk profile within the cohort. Most participants had only one examination in total (56%). *Smoking* and *stress level* were evaluated via subjective assessment by the participants. Smoking status comprised the categories of “never smoker,” “previous smoking,” and “current smoking.” For the assessment of stress levels, it was possible to choose between “low,” “moderate,” and “high” stress. *Arterial hypertension* was defined by either systolic blood pressure above 140 mm Hg or diastolic blood pressure above 90 mm Hg. Elevated low-density lipoprotein (LDL) was checked against the threshold of 130 mg/dL.^12^ *Obesity* was defined as a body mass index (BMI) >30 kg/m^2^, and *diabetes* as hemoglobin A1c (HbA1c) >6.5%.^13^ For better understanding of the global risk across the age groups in women and men, we calculated the SCORE 2.^14^ The Score 2 allows the 10-year risk of fatal and nonfatal CV events to be estimated in people under the age of 70, in accordance with current guidelines, and has been validated in large European cohorts.^4^^,^^14^ The SCORE 2 is a sex-specific model including the risk factors age, systolic blood pressure, gender, total cholesterol, high-density lipoprotein, and smoking status (yes/no).^14^

**Figure 1:**
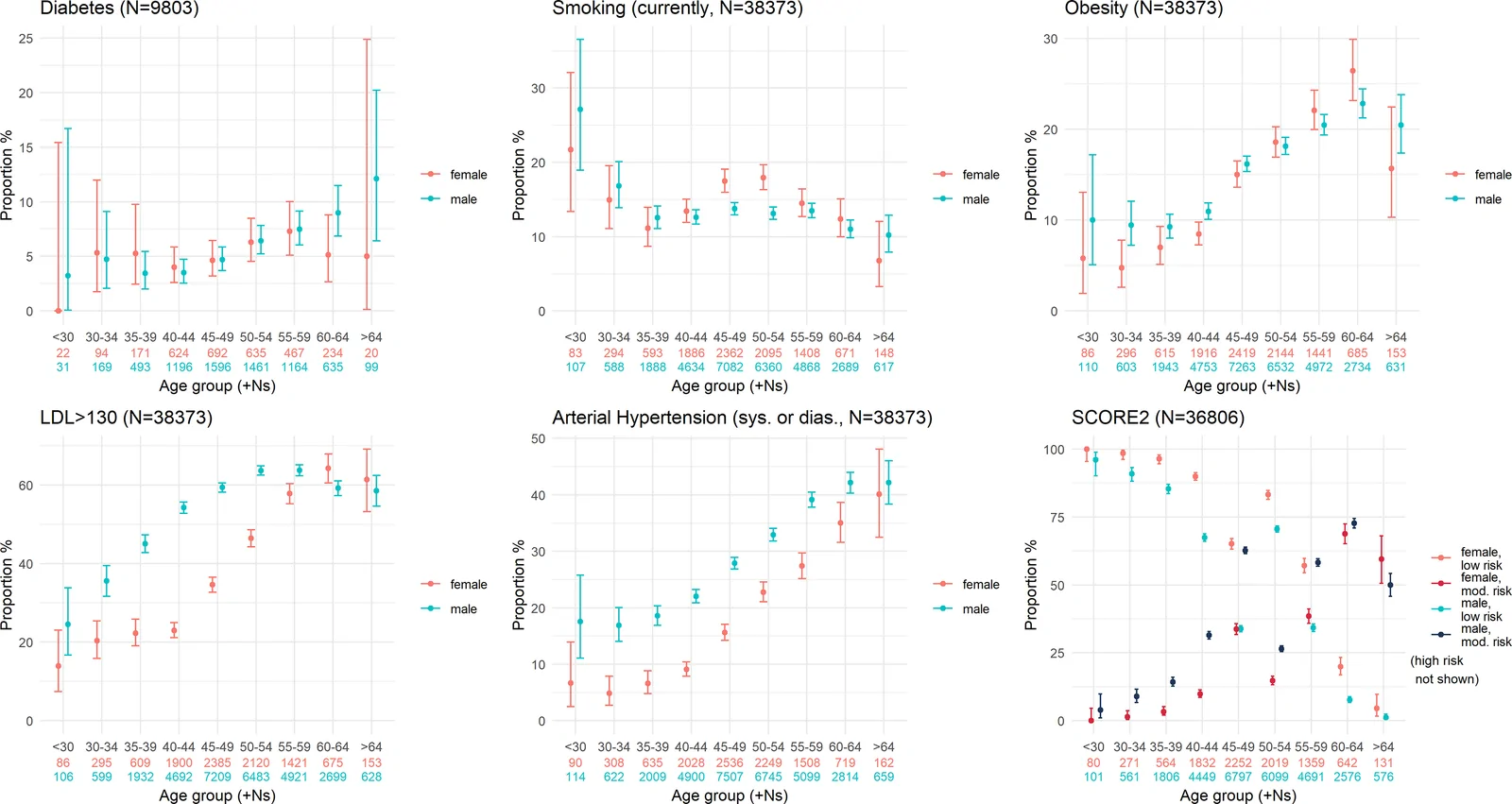
Sex- and Age-Specific Changes in Cardiovascular Risk Factors Changes in the prevalence of major cardiovascular risk factors and SCORE 2 within age groups of 5 years from <30 to >64 years in women and men. LDL = low-density lipoprotein.

In order to examine concisely sex- and age-specific differences, the cohort was divided into 6 groups: women <45 years (n = 3,061), women between 45 and 55 years (n = 5,139), and women >55 years (n = 2,035), as well as men <45 years (n = 7,645), men between 45 and 55 years (n = 15,396), and men >55 years (n = 7,428) (Table 1). Figure 1, on the other hand, gives a more fine-grained depiction of the CV risk factors in different age groups by splitting age into 9 5-year categories. In the following, we will focus mainly on the broader age categories for greater clarity.

**Table 1:** Patient Characteristics of the Study Population Overall and Stratified by Sex and Age

	Total (N = 40,704)	Female <45 (n = 3,061)	Female 45-55 (n = 5,139)	Female >55 (n = 2,035)	Male <45 (n = 7,645)	Male 45-55 (n = 15,396)	Male >55 (n = 7,428)
BMI (kg/m^2^)	26.3 (4.3)	23.6 (4.1)	25.5 (5.1)	26.7 (5.4)	25.7 (3.6)	26.8 (3.9)	27.5 (4.0)
Waist circ. (cm)	95 (12)	84 (11)	90 (13)	94 (13)	94 (10)	97 (11)	100 (11)
Body fat (tanita, %)	25 (8)	29 (7)	32 (8)	35 (7)	20 (6)	22 (6)	24 (6)
Blood pressure, sys. (mm Hg)	125 (16)	114 (14)	121 (16)	128 (17)	123 (13)	126 (15)	131 (16)
Blood pressure, dia. (mm Hg)	81 (9)	75 (8)	78 (9)	81 (9)	80 (8)	83 (9)	84 (9)
Intima-media thickness (mm)	0.61 (0.13)	0.52 (0.09)	0.59 (0.11)	0.66 (0.13)	0.55 (0.10)	0.62 (0.12)	0.70 (0.15)
Total cholesterol (mg/dL)	209 (40)	189 (33)	207 (38)	227 (41)	201 (39)	213 (40)	213 (41)
LDL (mg/dL)	134 (36)	109 (29)	125 (34)	142 (36)	131 (35)	141 (35)	140 (35)
HDL (mg/dL)	59 (20)	70 (24)	70 (22)	71 (19)	54 (18)	55 (17)	56 (20)
Triglycerides (mg/dL)	121 (76)	84 (43)	96 (52)	111 (58)	118 (74)	132 (81)	136 (84)
HbA1c (%)	5.39 (0.55)	5.15 (0.49)	5.32 (0.50)	5.53 (0.60)	5.26 (0.48)	5.41 (0.53)	5.57 (0.63)
CRP (mg/L)	0.73 (1.59)	1.06 (2.10)	0.99 (2.06)	0.75 (1.58)	0.73 (1.51)	0.63 (1.36)	0.62 (1.44)
HS-CRP (mg/L)	0.58 (1.70)	1.24 (2.54)	0.77 (2.01)	0.47 (1.40)	0.69 (1.76)	0.46 (1.43)	0.40 (1.59)
Smoking n (%)							
Ex-smoking	8,791 (23%)	503 (18%)	1,009 (21%)	466 (25%)	1,422 (20%)	3,328 (23%)	2,063 (29%)
Nonsmoker	24,341 (63%)	1,972 (69%)	2,934 (61%)	1,189 (63%)	4,845 (67%)	9,245 (64%)	4,156 (59%)
Smoker	5,241 (14%)	381 (13%)	849 (18%)	237 (13%)	950 (13%)	1,971 (14%)	853 (12%)
Stress (score) n (%)							
Low	4,429 (12%)	348 (12%)	626 (14%)	361 (20%)	634 (8.9%)	1,377 (9.7%)	1,083 (16%)
Moderate	19,849 (53%)	1,554 (56%)	2,639 (58%)	1,059 (59%)	3,836 (54%)	7,210 (51%)	3,551 (53%)
High	12,836 (35%)	886 (32%)	1,292 (28%)	371 (21%)	2,633 (37%)	5,548 (39%)	2,106 (31%)

Patient characteristics of the overall study population as well as stratified by sex and age (<45, 45-55, >55 years). Continuous variables are presented as mean (SD), categorical variables are presented as n (%).

BMI = body mass index; CRP = C-reactive protein; dia. = diastolic; HbA1c = hemoglobin A1c; HS-CRP = high-sensitivity CRP; HDL = high-density lipoprotein; LDL = low-density lipoprotein; n = number of nonmissing values; sys. = systolic; waist circ. = waist circumference.

### Statistical analysis

Due to the descriptive nature of this study, we report mean and SD and median and IQR, as well as proportions. All descriptives are accompanied by 95% CIs (binomial method for both medians and proportions). We deliberately decided against significance tests, as they are of very limited use in very large cohorts like ours. The amount of missing data is indicated in Tables and Figures; we refrained from imputation methods due to the descriptive nature of the analysis.

### Ethics

The study was reviewed and granted approval by the ethics committee of the Charité-Universitätsmedizin (EA4/077/24). The data set was transmitted to Charité in anonymized and encrypted form by ias PREVENT. The process of anonymization was carried out by ias PREVENT.

## Results

A total of 40,704 initial examinations were included in this analysis. Most examinations took place between 2011 and 2015 (55%, 22,384/40,704). The average age of the cohort was 49 years. Age refers to the participants’ age at the time of the initial visit. Overall, 25% (n = 10,235) were female while 75% (n = 30,469) were male.

### Women and men <45 years

The mean BMI was 22.8 kg/m^2^ in women and 25.2 kg/m^2^ in men. The mean waist circumference was 83 cm in women and 93 cm in men. Total cholesterol in women was 187 mg/dL, and LDL cholesterol was 107 mg/dL. In men, total cholesterol was 199 mg/dL and LDL cholesterol was 129 mg/dL. The average HbA1c level was 5.2% for women and 5.3% for men; blood pressure was 110/75 mm Hg for women and 120/80 mm Hg for men. The prevalence for hypertension was 8.1% (247/3,061; 95% CI: 7.1%-8.1%) in women and 21% (1,578/7,645; 95% CI: 20%-22%) in men. Elevated LDL was found in 22% (645/2,890; 95% CI: 21%-24%) of women and 50% (3,657/7,329; 95% CI: 49%-51%) of men, and diabetes in 4.3% (39/911; 95% CI: 3.1%-5.9%) of women and 3.6% (68/1,889; 95% CI: 2.8%-4.6%) of men, respectively.

The mean intima-media thickness (IMT) was 0.5 mm in women and 0.54 mm in men. Five hundred and three (18%) women stated that they were ex-smokers, compared to 1,422 (20%) of men. There were 1,972 (69%) of women and 4,845 (67%) of men who reported never having smoked. Thirteen percent of both sexes (381/3,061 women and 950/7,645 men) stated that they currently smoked. The highest number of current smokers was found among participants under 30 years of age in both sexes, with a lower number of smokers in the 30 to 45 age group.

In the subjective assessment of their own stress levels, 12% of women (348/3,061) reported low stress levels, 56% (1,554/3,061) moderate, and 32% (886/3,061) high stress levels. Among men, 8.9% (634/7,645) rated their stress levels as low, 54% (3,836/7,645) rated as moderate, and 37% (2,633/7,645) as high.

### Women and men between 45 and 55 years

Generally, measurements worsened slightly for both sexes compared to younger individuals. Indicators of obesity increased (women: average BMI of 24.4 kg/m^2^ and waist circumference 88 cm, men: BMI 26.2 kg/m^2^ and waist circumference 96 cm) (Table 1). Likewise, values of lipid and glucose metabolism worsened (women: total cholesterol 205 mg/dL, LDL cholesterol 123 mg/dL, HbA1c 5.3%; men: total cholesterol 211 mg/dL, LDL cholesterol 139 mg/dL, HbA1c 5.4%). Average blood pressure slightly increased to 120/80 mm Hg for women and 124/80 mm Hg for men. Hypertension was present in 19% (994/5,139; 95% CI: 18%-20%) of women and 31% (4,738/15,396; 95% CI: 30%-32%) of men, elevated LDL in 41% (1,991/4,844; 95% CI: 40%-43%) and 62% (9,130/14,798; 95% CI: 61%-62%), and diabetes in 5.2% (75/1,431; 95% CI: 4.2%-6.6%) and 5.6% (184/3,305; 95% CI: 4.8%-6.4%), respectively.

IMT averaged 0.59 mm in women and 0.6 mm in men. As for smoking habits, 21% (1,009/5,139) of women and 23% (3,328/15,396) of men identified as former smokers, while 61% (2,934/5,139) of women and 64% (9,245/15,396) of men had never smoked. Current smoking was reported by 18% (849/5,139) of women and 14% (1,971/15,396) of men. After the peak among active smokers under the age of 30, the number of active female smokers in the 45 to 55 age group was again higher, while the numbers for male smokers remained largely unchanged in this age group.

Self-reported stress levels showed that 14% (626/5,139) of women experienced low stress, 58% (2,639/5,139) moderate stress, and 28% (1,292/5,139) high stress. In men, the corresponding figures were 9.7% (1,377/15,396), 51% (7,210/15,396), and 39% (5,548/15,396), respectively.

### Women and men >55 years

Overall, cardiometabolic parameters further deteriorated in both sexes compared to the 45 to 55 age group. Measures of obesity increased, with women showing a mean BMI of 25.8 kg/m^2^ and a waist circumference of 93 cm, while men reached a mean BMI of 26.9 kg/m^2^ and a waist circumference of 99 cm (Table 1). Lipid parameters were less favorable, particularly in women (total cholesterol 223 mg/dL vs 212 mg/dL in men), whereas LDL cholesterol levels were comparable between sexes (139 mg/dL). Mean HbA1c levels remained stable overall at 5.5% in both women and men. Blood pressure increased modestly to an average of 125/80 mm Hg in women and 130/80 mm Hg in men. Among women, 32% (644/2,035; 95% CI: 30%-34%) had hypertension, 61% (1,170/1,910; 95% CI: 59%-63%) elevated LDL, and 7.1% (44/617; 95% CI: 5.3%-9.5%) diabetes; corresponding prevalences in men were 41% (3,038/7,428; 95% CI: 40%-42%), 61% (4,389/7,142; 95% CI: 60%-63%), and 8.5% (141/1,650; 95% CI: 7.3%-10%).

IMT values were higher than in younger individuals, averaging 0.65 mm in women and 0.70 mm in men. Regarding smoking behavior, the proportion of former smokers increased to 25% (466/2,035) among women and 29% (2,063/7,428) among men, while current smoking prevalence declined slightly (237 women [13%], 853 men [12%]). The majority of participants reported never having smoked (63% [1,189/2,035] of women and 59% [4,256/7,428] of men).

Perceived stress levels remained substantial in this age group. Among women, 20% reported low stress, 59% moderate stress, and 21% high stress. In men, stress levels were generally higher, with only 16% reporting low stress, 53% moderate stress, and 31% high stress.

### SCORE 2

After calculation of the SCORE2 for the different age groups, 92% (2,539/2,747) of women under 45 years showed a low and 7.4% (204/2,747) a moderate risk, while 74% (5,148/6,917) men showed a low, 25% a moderate, and 0.9% a high CV risk. The mean SCORE2 was 0.68 points for women and 2.07 points for men. In the group of women aged between 45 and 55 years, 73% (3,371/4,593) had a low, 25% (1,147/4,593) a moderate, and 1.6% (75/4,593) a high risk. Fifty-one percent (7,130/13,958) of men in the same age group had a low, 46% (6,373/13,958) a moderate, and 3.3% (455/13,958) a high risk. Women in this age group had a mean SCORE 2 of 1.83 points, men of 4.04 points. In the oldest age group, we found 38% (685/1,810) of women with low, 53% (954/1,810) with moderate, and 9.4% (171/1,810) with high risk. Nineteen percent (1,288/6,781) of men >55 years had a low, 65% (4,399/6,781) a moderate, and 16% (1,094/6,781) a high risk; the mean SCORE 2 for women was 4.25 points and for men 7.73 points (Figure 1).

### Sex differences in the prevalence of CV risk factors

When comparing the prevalence of individual CV risk factors, we observed a numerically higher prevalence of diabetes in men than in women aged 55 years and above, whereas in the group <40 years, the prevalence was higher in women than in men (Figure 1). Smoking prevalence in both sexes declined with increasing age. Of note, between the ages of 40 and 55 years, more women than men smoked. Concerning obesity, a higher prevalence was found in the older groups in both sexes, with an emphasis on women in the 50 to 60 years of age group. Furthermore, we saw differences in the prevalence of hyperlipoproteinemia, with a higher prevalence in men than in women already from the age of 30 years up to the age of 59 years (Figure 1). For hypertension, we observed a substantial higher prevalence in men than in women from the age of 30 to 64 years. A convergence in prevalence between the sexes was observed only in the age group above 64 years ( Figure 1). An overview of the results is summarized in the Central Illustration.

**Central Illustration:**
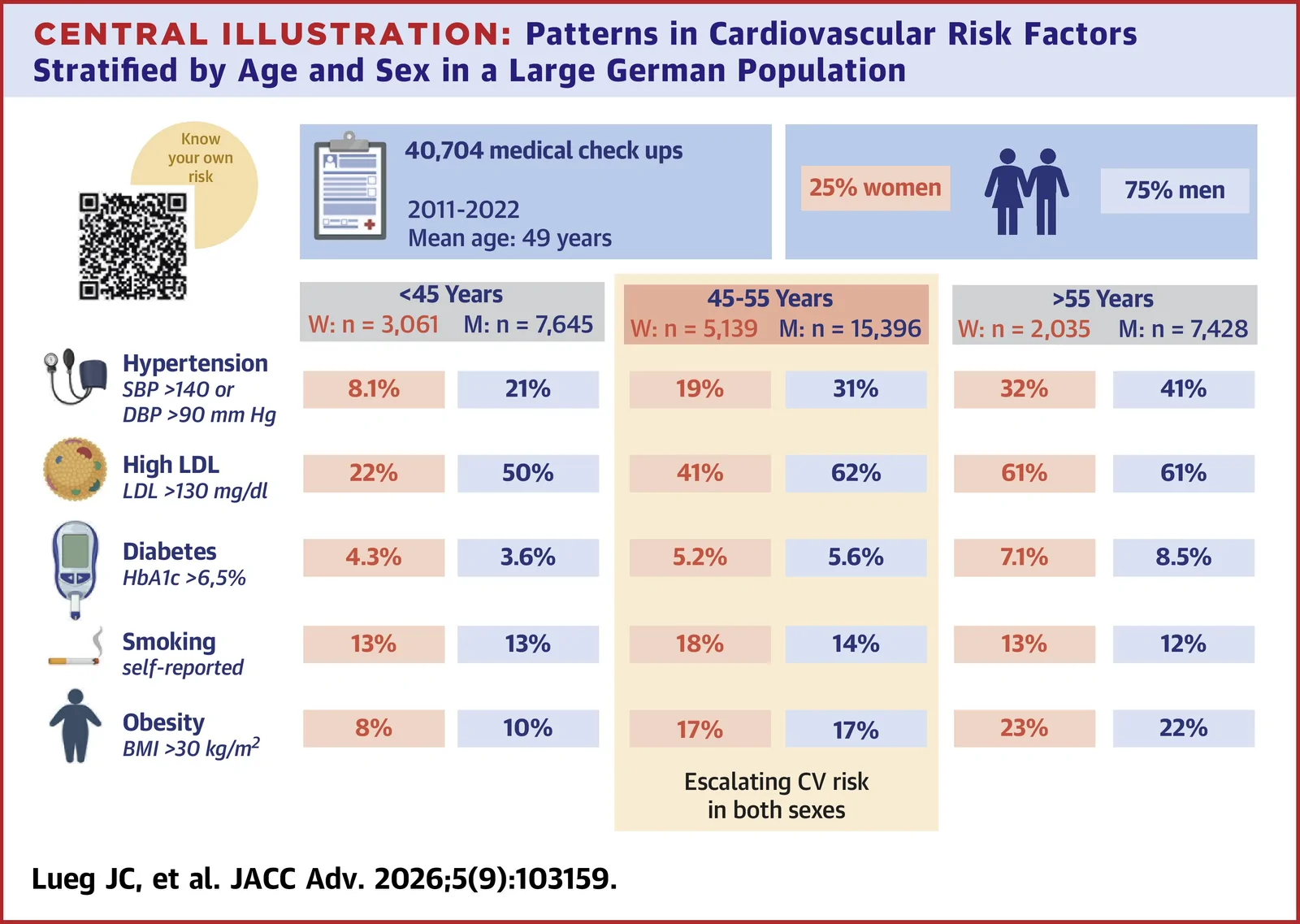
Patterns in Cardiovascular Risk Factors Stratified by Age and Sex in a Large German Population Data from 40,704 medical checkups of employees were analyzed stratified by sex and age. The cohort was mainly male (75%), mean age was 49 years. Prevalence of major cardiovascular risk factors including smoking, hyperlipidemia, hypertension, obesity, and diabetes was examined. An increase in cardiovascular risk factors occurred in men and women mainly between 45 and 55 years. These findings highlight the chances of sex- but especially age-specific targeted cardiovascular prevention strategies. Created in BioRender, Lueg J (2026), https://BioRender.com/2a6fsom. BMI = body mass index; CV = cardiovascular; DPB = diastolic blood pressure; HbA1c = hemoglobin A1c; SPB = systolic blood pressure; other abbreviation as in Figure 1.

To facilitate clinical application of the data presented, we have created a website where users can explore CV risk-related variables by sex and age and benchmark individual patient data against our cohort: www.ibike-shine.charite.de/CardioNormGER.

## Discussion

In this study, we analyzed data from 40,704 health checkups conducted by ias PREVENT between 2011 and 2022 to obtain a detailed picture of sex- and age-specific CV risk patterns in a large cohort of the working population. To our knowledge, this represents the largest study of CV risk patterns in the working population in Germany to date. The major strength of the cohort lies in the large number of individuals examined and the quantity and breadth of data available for evaluation.

The population studied was characterized by a low burden of CV risk factors. In the entire cohort, the values for mean BMI, systolic and diastolic blood pressure, IMT, and HbA1c were within normal ranges. However, exhibited elevated values for total cholesterol and LDL were observed. The changes of the CV risk with increasing age correspond to the findings within international, longitudinal studies like the Framingham study.^15^

### Age- and sex-specific prevalence of CV risk factors

Based on these data, CV risk increases with age in both men and women. Several interesting aspects emerged from the sex-specific and, above all, age-adjusted analysis. Observed CV risk patterns were mostly consistent with previous studies.^16^^,^^17^ In our cohort, the prevalence of arterial hypertension increased with age. Since this study focuses on the working population, age groups over 65 were not included, but the aforementioned trend toward an increase in arterial hypertension in women, especially in older age, was also evident in this cohort.^16^^,^^18^

Previous studies show that there are significant regional differences in the prevalence of smoking among men and women.^3^^,^^17^^,^^19^ This German cohort revealed a surprisingly high proportion of active female smokers, which was higher than in men in several age groups. Given the increased risk of CV events in women who smoke and use oral contraception,^20^ sufficient education about smoking as a risk factor should be further emphasized in preventive approaches. Data on the German population show a slightly different picture: more men than women smoke overall, including in the 45 to 55 age group. This therefore appears to be a distinctive feature of this age group.^21^

With regard to hyperlipoproteinemia, the values also showed an expected progression with a rapid increase in cholesterol levels in women from the age of 45.^19^ With the onset of menopause, on average at the age of 50 years,^22^ LDL concentrations rise in women with the decline in estrogen.^23^ Thus, younger women initially have more cardioprotective lipid profiles than men.^23^ We were able to show a similar temporal progression in this study for obesity in men and women. Here, too, the most significant increase in prevalence was observed from the age of 45 onward, coinciding with the potential time of perimenopause in women. Greendale et al^24^ demonstrated that the onset of the menopause transition in women is associated with a doubling of the rate of fat mass gain alongside a concurrent decline in lean mass. The menopause-related shifts in body composition may represent a key biological mechanism underlying the escalating CV risk profile observed in perimenopausal women. At the same time, the increase in body weight is also evident among men in this age group in our data. More detailed analyses of sex-specific differences in fat and muscle mass would be necessary in order to assess the resulting sex-specific CV risk. These data were not collected in the checkups analyzed.

Overall, this study highlights the particular relevance for CV risk factor changes at the age of 45 to 55 years in both men and women. When planning individualized prevention approaches, particular attention should be paid to this age group, as our data show the most pronounced changes in CV risk factors in an overall healthy cohort. This period coincides with the time frame in which menopausal transition typically occurs.^3^ Awareness of one's own CV risk and personal initiative to proactively control treatable risk factors could be explicitly addressed and improved. The menopausal transition may represent a valuable opportunity to raise CV risk awareness and promote proactive risk factor management in women.^1^^,^^3^

### Sex-specific differences in participation in health screenings

Consistent with the aforementioned findings, women again were underrepresented in this cohort. Where available, data on the uptake of medical checkups in the working environment paint a similar picture for women and men.^25^ Overall, there is a tendency for women to take advantage of regular cancer screenings and medical checkups more often than men.^26^ The disparity in this case may reflect several contributing factors. A relevant factor that determines perception and participation in health checkups could be communication and information regarding the available services. Due to the anonymization of this cohort and participating companies, no further details concerning underlying causes of the sex imbalance could be obtained.

### Health care system context in Germany

The German health care system is characterized by mandatory, employment-based insurance, predominantly provided through statutory health insurance, with optional private coverage for higher-income groups. The ias PREVENT examinations analyzed in this study were independent, voluntary, employer-supported checkups neither part of routine care nor statutory health insurance. The checkups were mainly offered to employees in management positions and executive roles. The selective recruitment introduces a phenomenon called the healthy worker effect as such individuals are generally healthier and more engaged with preventive care.^27^^,^^28^ Accordingly, the results should be interpreted in this context, and limited comparability to unemployed or retired populations should be considered.

### Bridging gaps in workplace health promotion

Although overall working conditions and general occupational safety have improved over the past centuries, several challenges remain, and new ones have emerged. The rising prevalence of CV diseases, partly attributed to high stress levels, increasingly sedentary occupations, and unhealthy dietary choices, has prompted occupational health and safety specialists to call for new measures of prevention focusing on workplace-related behaviors.^29^^,^^30^ According to the German Federal Institute for Occupational Safety and Health (Bundesanstalt für Arbeitsschutz und Arbeitsmedizin, BAuA), CV diseases represent one of 2 leading causes for inability to work in Germany.^31^ Against this background, the occupational framework offers great potential to actively promote and implement health initiatives targeting CV diseases and other major public health challenges. Health screenings, easy access to vaccinations, workplace mobility programs, as well as offering healthy dietary options, bear great potential in reducing the public health burden. The Luxembourg Declaration on Workplace Health Promotion (WHP) in the European Union defines WHP as a joint effort of employers, employees, and society.^32^^,^^33^ WHP measures are diverse and include f.e. analyses and surveys on health status, exercise programs, preventive programs against addictions, and individual health checks.^32^^,^^33^ Recent data suggest that in Germany approximately half of all employees have access to WHP measure; however, comprehensive and multicomponent options remain scarcely offered. Importantly, implementation is reported to be strongly associated with company size and economic status.^32^^,^^34^

In summary, these data underline the necessity, urgency, and potential of improving WHP both quantitatively and qualitatively. Women, often working in critical infrastructure and essential occupations, represent a constantly underrepresented and therefore overlooked but crucial target for workplace prevention. Our study highlights the importance of the age period of 45 to 55 years in both men and women.

### Study Limitations

Despite the substantial size of the cohort with checkups from the last decade, it was not designed to obtain a representative number of certain parameters such as CV endpoints (eg, myocardial infarction, heart failure, hospitalization, or death). The reappraisal of missing values was rendered unfeasible due to the constraints imposed by the company's data protection policies. Therefore, sex-specific and novel risk factors like history of adverse pregnancy outcomes, polycystic ovary syndrome, or Lipoprotein(a) could not be included in the analysis. Furthermore, the analysis includes checkups from the past 10 years, when awareness of such risk factors was still low. The various documentation of measures across different locations resulted in a comparatively high number of missing values.

As participants entered the cohort at different time points, age at the first visit is inherently confounded by the timing of the medical checkup. Observed differences in sex-specific CV risk may partly reflect temporal trends in risk factor prevalence, diagnostic criteria, or treatment standards rather than true age-related differences. Furthermore, the composition of sexes in the cohort represents a significant limitation, as the proportion of women was low at 25%, which may have limited the precision of sex-specific estimates.

Information concerning the patient's current medication history, preventive pharmacotherapy, and previous illnesses was absent. Therefore, it was only possible to draw approximate conclusions regarding the women’s premenopausal, perimenopausal, and postmenopausal status based on their age, and no causal link can, therefore, be established. Moreover, the data set did not include information regarding the participants' current job positions. Consequently, the results of the subjectively assessed stress level were largely noncomparable in terms of sex and age. A potential bias should be acknowledged, given that the cohort comprises exclusively employed individuals who likely benefit from above-average health care access and preventive screenings. This may have contributed to the overall low burden of CV risk factors in the cohort and limit the generalizability of the key cardiometabolic parameters to broader, nonworking, or retired populations.

## Conclusions

This study analyzed the largest German cohort in the working population to date with regard to existing age- and sex-specific CV risk. Despite the overall low burden of CV risk factors, an escalation in CV risk was observed among participants, especially in the age group of 45 to 55 years. As this period coincides with perimenopause in most women, it could be used as a window of opportunity to raise awareness about their CV health. Sex-specific campaigns could therefore lead to individualized improvements in CV health in the working population.

Perspectives **COMPETENCY IN MEDICAL KNOWLEDGE:** CV risk profiles differ slightly between women and men across age groups, with escalating risk during the period between 45 and 55 years. Sex- and age-specific risk assessment with heightened screening intensity during this time period should be integrated into routine clinical practice. Individual patient values can be compared with those of the cohort in this study as follows: http://www.ibike-shine.charite.de/CardioNormGER.

**TRANSLATIONAL OUTLOOK:** Longitudinal studies should evaluate workplace-centered prevention strategies on long-term CV outcomes.

## Funding support and author disclosures

Dr Collisi is a senior physician at ias PREVENT Berlin and a member of the management board, while Dr Stangl is a board member of the ias Foundation. The study was funded by ias PREVENT with €15,000 over 3 years. All other authors have reported that they have no relationships relevant to the contents of this paper to disclose.
